# Comparing Contact Time in Traditional vs. Relational Autonomy Approaches in Mental Health Practice

**DOI:** 10.1192/j.eurpsy.2026.11654

**Published:** 2026-07-31

**Authors:** I. Sohn, I. Cho

**Affiliations:** 1 Psychiatry, Keyo Hospital, Uiwang; 2 WIM psychiatric clinic, Seongnam, Korea, Republic Of

## Abstract

**Introduction:**

Autonomy has long been regarded as a cornerstone of ethical mental health practice. However, the way autonomy is framed shapes the therapeutic relationship and resource allocation. While traditional autonomy emphasizes an individual’s capacity for independent decision-making, relational autonomy highlights the social and interpersonal dimensions that influence choices. Understanding how these frameworks translate into practical differences in clinical encounters is crucial for both ethical deliberation and service planning.

**Objectives:**

This study aims to compare the contact time clinicians spend with patients and families under traditional autonomy and relational autonomy approaches in mental health practice.

**Methods:**

51 mental health professionals participated in an online survey. Each respondent estimated average time spent per encounter with a patient and family caregiver when applying traditional autonomy and relational autonomy approaches, using the scenario of an acutely psychotic patient with schizophrenia.

**Results:**

Under the traditional autonomy approach, clinicians reported spending an average of 10.21 minutes each with the patient and the family. Under the relational autonomy approach, these times increased to 17.86 and 15.36 minutes, respectively—an increase of approximately 7 to 8 minutes. Respondents noted that relational autonomy requires more time for empathic dialogue and collaborative explanation.

**Conclusions:**

The choice of autonomy framework significantly affects clinical time expenditure. Relational autonomy fosters richer patient interactions but requires more resources, highlighting implications for staffing and clinical workflow in mental health services.

**Disclosure of Interest:**

None Declared

